# XBB.1.5 COVID-19 mRNA Vaccines Induce Inadequate Mucosal Immunity in Patients with Inflammatory Bowel Disease

**DOI:** 10.3390/vaccines13070759

**Published:** 2025-07-16

**Authors:** Simon Woelfel, Joel Dütschler, Daniel Junker, Marius König, Georg Leinenkugel, Claudia Krieger, Samuel Truniger, Annett Franke, Seraina Koller, Katline Metzger-Peter, Nicola Frei, STAR SIGN Study Investigators, Werner C. Albrich, Matthias Friedrich, Jan Hendrik Niess, Nicole Schneiderhan-Marra, Alex Dulovic, Wolfgang Korte, Justus J. Bürgi, Stephan Brand

**Affiliations:** 1 Department of Gastroenterology and Hepatology, HOCH, Cantonal Hospital St. Gallen, 9007 St. Gallen, Switzerland; 2 Max von Pettenkofer Institute of Hygiene and Medical Microbiology, Faculty of Medicine, Ludwig Maximilian University (LMU), 80333 Munich, Germany; 3 Translational Gastroenterology and Liver Unit, Nuffield Department of Medicine, University of Oxford, Oxford OX3 9DU, UK; 4 Outpatient Clinic, HOCH, Ambulatory Services Rorschach, 9400 Rorschach, Switzerland; 5 NMI Natural and Medical Sciences Institute at the University of Tübingen, 72770 Reutlingen, Germany; 6 Department of Gastroenterology and Hepatology, University Digestive Healthcare Center, Clarunis, 4002 Basel, Switzerland; 7 Division of Infectious Diseases, Infection Prevention & Travel Medicine, HOCH, Cantonal Hospital St. Gallen, 9007 St. Gallen, Switzerland; 8 Gastroenterology Group, Department of Biomedicine, University of Basel, 4031 Basel, Switzerland; 9 Center for Laboratory Medicine, 9001 St. Gallen, Switzerland

**Keywords:** mucosal immunity, COVID-19, inflammatory bowel disease, anti-TNF, mRNA vaccines, SARS-CoV-2, XBB.1.5, JN.1, IgA

## Abstract

Background: Mucosal immunity plays a pivotal role in preventing infections with SARS-CoV-2. While COVID-19 mRNA vaccines induce robust systemic immune responses in patients with inflammatory bowel disease (IBD), little is known about their efficacy in the mucosal immune compartment. In this sub-investigation of the ongoing STAR-SIGN study, we present the first analysis of mucosal immunity elicited by XBB.1.5 mRNA vaccines in immunocompromised patients with IBD. Methods: IgG and IgA antibodies targeting the receptor-binding domain of the SARS-CoV-2 JN.1 variant were quantified longitudinally in the saliva of IBD patients using the multiplex immunoassay MultiCoV-Ab. Antibody levels were quantified before and 2–4 weeks after vaccination with XBB.1.5 mRNA vaccines. All patients previously received three doses with original COVID-19 vaccines. Results: Mucosal IgG antibodies were readily induced by XBB.1.5 mRNA vaccines (*p* = 0.0013 comparing pre- and post-vaccination levels). However, mucosal IgA levels were comparable before and after vaccination (*p* = 0.8233). Consequently, mucosal IgG and IgA antibody levels correlated only moderately before and after immunization (pre-vaccination: *r* = 0.5294; *p* = 0.0239; post-vaccination: *r* = 0.4863; *p* = 0.0407). Contrary to a previous report in healthy individuals, vaccination did not induce serum IgA in patients with IBD (*p* = 0.5841 comparing pre- and post-vaccination levels). These data suggest that COVID-19 mRNA vaccines fail to elicit mucosal IgA in patients with IBD. Conclusions: Since mucosal IgA plays a pivotal role in infection control, the lack of IgA induction indicates that patients lack sufficient protection against SARS-CoV-2 infections which warrants the development of mucosal COVID-19 vaccines.

## 1. Introduction

COVID-19 mRNA vaccines established the novel mRNA technology in mainstream healthcare and initiated a new era of vaccine development [1]. A key benefit of mRNA vaccines is their rapid adaptability to fast-evolving pathogens [2]. During the COVID-19 pandemic, this enabled their tailoring to novel SARS-CoV-2 variants which evolved in a way that allows them to escape immune responses elicited by previous vaccines readily [3,4,5]. Vaccination with mRNA vaccines robustly induces variant-specific neutralizing antibodies and continues to save lives [6]. Additionally, COVID-19 vaccines remain the only effective prevention measure for post-acute sequelae of COVID-19—often referred to as long COVID [7,8]. Therefore, COVID-19 mRNA vaccines remain crucial in preventing COVID-19 mortality and protecting individuals with an elevated risk of infectious diseases. Patients with immunosuppressive disease frequently mount insufficient immune responses following infection or vaccination, necessitating detailed immune status monitoring in these patients [9,10].

Inflammatory bowel diseases (IBD) are multifactorial immune disorders with a high prevalence that affect the gastrointestinal tract and often require immunosuppressive therapy [11,12,13,14]. Certain therapies commonly used to treat IBD have been shown to impair the immunogenicity of COVID-19 vaccines, including reduced levels of systemic IgG and neutralizing antibodies [15,16,17,18,19,20,21]. Consequently, patients with IBD receiving such treatments are at elevated risk for SARS-CoV-2 infection [22,23].

We recently reported that immunocompromised patients with IBD rely on variant-adapted COVID-19 mRNA vaccines to mount systemic immunity against the antigenically distinct and highly immune-evasive SARS-CoV-2 JN.1 variant [24,25]. Patients exclusively vaccinated with original mRNA vaccines lacked neutralization against JN.1 and other omicron lineages, undermining the importance of continuous booster immunization [24,26]. In recent years, however, even vaccine adaptation failed to prevent seasonal COVID-19 surges, and current mRNA vaccines seem to be suboptimal in blocking the transmission of ever-adapting SARS-CoV-2 variants. Early IgA responses at mucosal virus entry sites emerge as a critical determinant for infection control and clearance, indicating that interventions boosting mucosal IgA may prevent SARS-CoV-2 variant transmission [27,28]. However, recent studies evaluating COVID-19 mRNA vaccine-elicited mucosal immunity in healthy individuals reached conflicting conclusions, highlighting the need for further investigation [29,30].

In this sub-investigation of the STAR SIGN study, we quantified mucosal IgG and IgA targeting the SARS-CoV-2 JN.1 variant receptor-binding domain (RBD) in immunocompromised patients with IBD before and after immunization with XBB.1.5 mRNA vaccines.

## 2. Materials and Methods

### 2.1. Study Design and Patient Recruitment

Participants included in this analysis were recruited within the STAR SIGN (**S**ystemic and **T**-cell-**a**ssociated **r**esponses to **S**ARS-CoV-2 **i**mmunization in **g**ut i**n**flammation) study, a prospective multi-center cohort study investigating COVID-19 mRNA vaccine responses in patients with IBD [19,24,26,31]. The local ethics board (Ethikkommission Ostschweiz; EKOS) approved the protocol of the STAR SIGN study under the project ID 2021-02511. Patients were recruited at three tertiary IBD centers in Switzerland, including Cantonal Hospital St. Gallen, Ambi Rorschach, and Digestive Healthcare Center Clarunis Basel. Participants were recruited during routine hospital visits. The treating physician identified suitable patients. Patients were provided with detailed study information and were given sufficient time to consider participation in the study carefully. Adult patients with ulcerative colitis, Crohn’s disease, or indeterminate colitis, receiving advanced therapy (biologics or small molecule inhibitors), who were triple-vaccinated with original COVID-19 mRNA vaccines (BNT162b2 by BioNTech/Pfizer or mRNA-1273 by Moderna), were considered. Only patients who reported that they were not SARS-CoV-2-infected and not vaccinated against COVID-19 at any time within six months before the start of the study were eligible for study inclusion. Patients were not included in the study if they received treatment with steroids, immunomodulators, or checkpoint inhibitors at any time within six months before study start. Additionally, study participants who reported a COVID-19 infection at any time during study participation were excluded from the study. All participants provided written informed consent before their enrollment in the study.

### 2.2. Study Procedures

The study procedures were performed during two visits by trained healthcare professionals at the participating study sites. The two study visits were two to four weeks apart, depending on the scheduled routine visits of participants. A minimum of two weeks between study visits was chosen to allow sufficient time for immune response build-up following vaccination. During study visit 1, saliva was collected, and participants were vaccinated with BNT162b2 XBB.1.5 (BioNTech/Pfizer) or mRNA-1273.815 (Moderna), both directed at the XBB.1.5 variant, based on their preference. During study visit 2, saliva collection was repeated. Consequently, saliva collection timepoints were before (=the day of) and two to four weeks after immunization with XBB.1.5 COVID-19 mRNA vaccines.

### 2.3. Quantification of Mucosal Anti-Receptor-Binding Domain Antibodies

Antibody quantification was performed at the Natural and Medical Sciences Institute at the University of Tübingen. IgG and IgA antibodies targeting the RBD of the SARS-CoV-2 JN.1 variant were quantified in saliva using MultiCoV-Ab, a bead-based multiplex immunoassay [32]. Method details on quantifying antibodies in saliva using MultiCoV-Ab were published previously [26].

### 2.4. Study Outcomes and Statistical Analysis

The primary outcomes were SARS-CoV-2 JN.1 variant-specific anti-RBD IgG and IgA levels in saliva from patients with IBD. Analysis timepoints were the day of (=before) and two to four weeks after immunization with XBB.1.5 mRNA vaccines as a fourth vaccine dose. The secondary outcomes were the correlation between IgG and IgA antibodies in saliva before and after vaccination, and levels of serum IgA post-vaccination.

The primary and secondary outcomes were analyzed using exact Wilcoxon signed-rank tests where applicable, and the secondary outcomes were additionally analyzed using linear regression analyses and Spearman’s rank correlations.

Statistical analyses were performed using GraphPad Prism, version 10.5.0.

## 3. Results

### 3.1. Patient Characteristics

Eighteen patients with a mean age of 49.3 years (standard deviation [SD] 16.8 years) were included. Patients were diagnosed with ulcerative colitis (55.6%), Crohn’s disease (38.9%), or indeterminate colitis (5.6%) and were treated with infliximab (61.1%), vedolizumab (27.8%), ustekinumab (5.6%), or tofacitinib (5.6%). All patients had received three original COVID-19 mRNA vaccine doses and were neither SARS-CoV-2-vaccinated nor-infected six months before study inclusion. None of the participants were treated with steroids, immunomodulators, or checkpoint inhibitors six months before their study inclusion. Detailed study population characteristics were published previously [24].

### 3.2. XBB.1.5 mRNA Vaccines Do Not Boost Mucosal IgA in Patients with IBD

**Figure 1:**
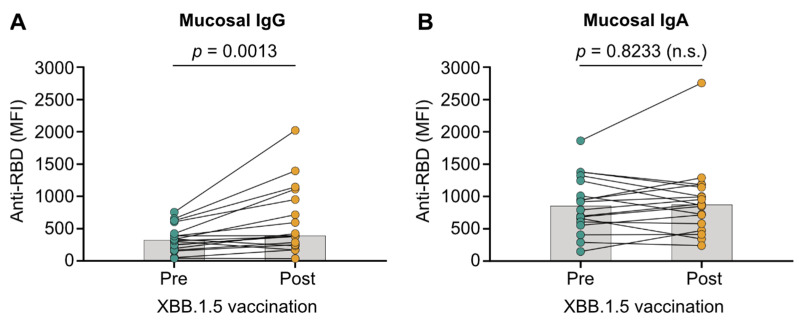
SARS-CoV-2 JN.1 variant-targeting mucosal immunity elicited by variant-adapted COVID-19 mRNA vaccines. Anti-receptor-binding domain (RBD) IgG (**A**) and IgA (**B**) levels in saliva of patients with IBD presented as mean fluorescence intensity (MFI). Samples were collected before (Pre) and two to four weeks after (Post) receiving a fourth vaccine dose with XBB.1.5 mRNA vaccines. Median values are indicated by bars. Statistical analyses are based on exact Wilcoxon signed-rank tests.

We assessed mucosal anti-JN.1 immunity before and after immunization with XBB.1.5 mRNA vaccines to test if vaccination induces mucosal IgA, necessary for adequate SARS-CoV-2 transmission blocking [27,28]. Saliva anti-RBD IgG levels were higher after XBB.1.5 vaccination than before (*p* = 0.001; Figure 1A). These findings indicate that mRNA vaccines efficiently induce mucosal IgG. However, IgA levels were comparable before and after immunization (*p* = 0.823; Figure 1B), indicating no IgA induction by immunization. While levels of mucosal IgA were comparable between patients treated with infliximab and vedolizumab, infliximab-treated patients had reduced levels of mucosal IgG after vaccination (Supplementary Figure S1; IgA: *p* > 0.9999; IgG: *p* = 0.0147). Interestingly, the only patients who did not have higher mucosal IgG levels post- compared to pre-vaccination received infliximab treatment (n = 3; Figure 1A). Levels of mucosal IgA and IgG were comparable between patients diagnosed with Crohn’s disease and ulcerative colitis (Supplementary Figure S2; IgA: *p* = 0.0553; IgG: *p* = 0.6868).

**Figure 2:**
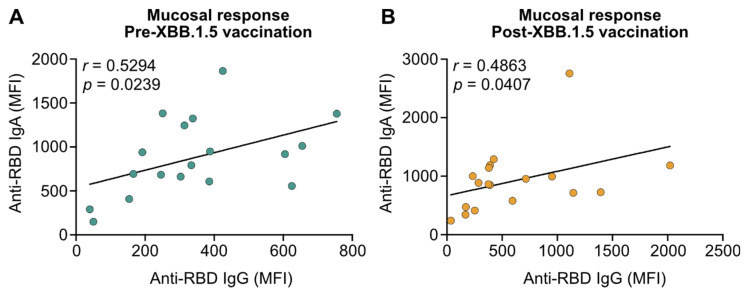
Correlation of SARS-CoV-2 JN.1 variant-targeting mucosal IgG and IgA before and after immunization with XBB.1.5 mRNA vaccines. Correlation of anti-receptor-binding domain (RBD) IgG and IgA levels in saliva from patients with IBD before (Pre; (**A**)) and two to four weeks after (Post; (**B**)) receiving a fourth vaccine dose with XBB.1.5 mRNA vaccines. Statistical analyses are based on Spearman’s rank correlations.

Mucosal IgG and IgA antibody levels moderately correlated before immunization (*r* = 0.5294; *p* = 0.0239; Figure 2A). After vaccination, IgG and IgA antibody levels still only showed a moderate correlation (*r* = 0.4863; *p* = 0.0407; Figure 2B). IgG levels were increased but IgA levels were comparable, resulting in a lower r value post- compared to pre-immunization (*r* = 0.5294 pre-vaccination vs. *r* = 0.4863 post-vaccination; Figure 2A,B). The absence of a strong correlation between mucosal IgA and IgG suggests that they are differentially affected by mRNA vaccination, emphasizing that only mucosal IgG but not the dominant mucosal immunoglobulin IgA are induced by vaccination. Given the critical role of anti-viral IgA in infection control upon mucosal entry of SARS-CoV-2 variants, the lack of IgA induction potentially renders COVID-19 mRNA vaccines ineffective in blocking virus variant transmission.

### 3.3. Migration of Vaccine-Induced Systemic IgA to the Respiratory Mucosa Is Absent in Patients with IBD

**Figure 3:**
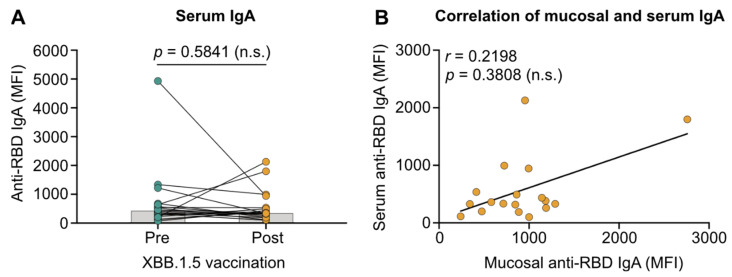
SARS-CoV-2 JN.1 variant-targeting systemic immunity elicited by variant-adapted COVID-19 mRNA vaccines and its correlation with mucosal immunity. (**A**) Anti-receptor-binding domain (RBD) IgA levels in the serum of patients with IBD are presented as mean fluorescence intensity (MFI). Samples were collected before (Pre) and two to four weeks after (Post) receiving a fourth vaccine dose with XBB.1.5 mRNA vaccines. Median values are indicated by bars. (**B**) Correlation of anti-RBD IgA levels in saliva and serum from patients with IBD post-vaccination. Statistical analyses are based on the exact Wilcoxon signed-rank test (**A**) and Spearman’s rank correlation (**B**).

A recent study suggested that repeated vaccination with mRNA-based vaccines increases mucosal IgA levels via induction of serum IgA and migration to the respiratory mucosa [26]. Since we did not observe increased mucosal IgA levels in our IBD patient cohort following vaccination, we sought to assess if this was due to missing systemic IgA induction. To this end, we quantified serum anti-RBD IgA levels and correlated them with mucosal IgA levels. Serum anti-RBD IgA levels were comparable before and after immunization with XBB.1.5 vaccines (*p* = 0.5841; Figure 3A). Furthermore, serum and mucosal anti-RBD IgA levels showed no correlation (*r* = 0.2198; *p* = 0.3808; Figure 3B). These findings suggest that mRNA-based vaccination does not induce systemic IgA and, consequently, does not contribute to mucosal immunity via IgA migration in patients with IBD.

## 4. Discussion

The continuous study of mRNA vaccines remains crucial to understand their strengths and pitfalls and to harness their full potential. In the post-omicron era of COVID-19, natural infections fail to sufficiently protect against SARS-CoV-2 reinfection due to sophisticated immune evasion mechanisms of circulating variants [33]. Therefore, vaccines that efficiently block the transmission of SARS-CoV-2 are crucial to protect at-risk individuals. Our findings suggest that current vaccines inadequately induce mucosal IgA levels in patients with IBD. Given that mucosal IgA plays a pivotal role in preventing SARS-CoV-2 infections upon virus exposure, this suggests that mRNA vaccines may fail to block virus transmission and to avoid SARS-CoV-2 infections [34,35,36].

Aligning with our results, a recent study showed that XBB.1.5 mRNA vaccines induce robust humoral and neutralizing immunity against the JN.1 variant in the peripheral immune system without sufficiently boosting IgA and respective neutralization in the mucosal compartment [29]. This aligns with our data on lacking IgA induction presented in this manuscript and with previously published data showing that vaccination induces robust humoral immunity including neutralizing IgG in the serum of the same patients assessed in the presented study [20]. In contrast, another study in triple-vaccinated individuals reported that original vaccines elicit mucosal IgA and respective neutralization [30]. Rather than stimulating the mucosal immune system, this was achieved by migrating systemic antibodies to the respiratory mucosa, which likely has limited efficacy in preventing SARS-CoV-2 infections. We recently demonstrated that intramuscularly administered COVID-19 vaccines fail to induce mucosa-residing IgA against several SARS-CoV-2 variants in patients with chronic liver disease and in the same IBD cohort assessed in the present study [31,37]. Combined with these studies, the presented analysis uncovers an impactful limitation of current COVID-19 mRNA vaccines and illustrates the essentiality of understanding vaccine-elicited immune responses in the mucosal compartment. Several studies have shown that anti-TNF-treated patients with IBD have impaired systemic IgG responses to COVID-19 vaccination [15,16,17,26]. Our comparison of mucosal immune responses between infliximab- and vedolizumab-treated patients suggests that this phenomenon is not limited to the systemic immune system but may also manifest at mucosal sites.

We acknowledge several limitations of our study. First, our study was not designed to evaluate vaccination efficacy. Given the growing body of research highlighting the importance of mucosal IgA in blocking COVID-19 infections, it seems plausible that low levels of virus-targeting mucosal IgA may not optimally block SARS-CoV-2 infections [27,34,35,36]. More research is needed to assess if boosting mucosal IgA in immunosuppressed patients with IBD can compensate for their increased infection susceptibility that was previously described [22,23]. Second, our study lacks the analysis of a healthy control group. Third, our study does not assess the long-term dynamics of the mucosal antibody response to COVID-19 vaccination. Third, our study is limited by its small sample size, which may affect the statistical power of subgroup analyses. High IgA levels in some study participants may reflect heterogeneous immune status, which requires validation in larger studies.

Notably, several studies have highlighted the great value of mRNA vaccines in preventing COVID-19-associated mortality [6,38,39]. The robust induction of systemic neutralizing antibodies and cellular immunity achieves vaccine-mediated protection from severe COVID-19. [40,41,42]. Therefore, mRNA vaccines remain a powerful tool for boosting immune responses and reducing the severity of breakthrough infections [43,44]. The mechanisms underlying mRNA vaccine-elicited immunity and how they can be exploited to optimize infection prevention are the subject of ongoing research [45,46]. An improved understanding and the continuous optimization of vaccination strategies are required to protect at-risk individuals and prevent the spread of SARS-CoV-2. Mucosal vaccines offer a promising approach to boost mucosal IgA and enhance protection against SARS-CoV-2 variants [47,48,49,50].

We advocate fostering their development to protect patients with IBD and, if approved, prioritize vaccination of anti-TNF-treated patients who have impaired immune responses to mRNA vaccines [19,24,26]. Targeted prevention of SARS-CoV-2 transmission can potentially reduce its spread and ultimately end the cycle of vaccine adaptation and the emergence of novel SARS-CoV-2 variants that overcome vaccine-elicited immunity.

## 5. Conclusions

This is the first study that investigates XBB.1.5 mRNA vaccine-elicited mucosal immunity against the SARS-CoV-2 JN.1 variant in patients with IBD. We show that vaccination induces mucosal IgG but not IgA. Mucosal IgG levels were lower in infliximab-treated patients compared to those threated with vedolizumab. We did not find evidence of IgA migration from systemic to mucosal sites. Collectively, our findings highlight an intrinsic weakness of mRNA vaccines and undermine the importance of exploring alternative immunization strategies, such as mucosal vaccines.

## Data Availability

The data presented in this study are available on request from the corresponding author due to ethical restrictions.

## Supplementary Materials

The following supporting information can be downloaded at: https://www.mdpi.com/article/10.3390/vaccines13070759/s1, Figure S1: SARS-CoV-2 JN.1 variant-targeting mucosal immunity following vaccination with variant-adapted COVID-19 mRNA vaccines, stratified by IBD therapy (VED: vedolizumab; IFX: infliximab). Anti-receptor binding domain (RBD) IgA (A) and IgG (B) levels in saliva of patients with IBD presented as mean fluorescence intensity (MFI). Samples were collected two to four weeks after receiving a fourth vaccine dose with XBB.1.5 mRNA vaccines. Median values are indicated by bars. Statistical analyses are based on exact Mann-Whitney tests; Figure S2: SARS-CoV-2 JN.1 variant-targeting mucosal immunity following vaccination with variant-adapted COVID-19 mRNA vaccines, stratified by IBD diagnosis (CD: Crohn’s disease; UC: ulcerative colitis). Anti-receptor binding domain (RBD) IgA (A) and IgG (B) levels in saliva of patients with IBD presented as mean fluorescence intensity (MFI). Samples were collected two to four weeks after receiving a fourth vaccine dose with XBB.1.5 mRNA vaccines. Median values are indicated by bars. Statistical analyses are based on exact Mann-Whitney tests; Table S1: STAR SIGN study investigators.
