# Supplementary material for: XBB.1.5 COVID-19 mRNA Vaccines Induce Inadequate Mucosal Immunity in Patients with Inflammatory Bowel Disease
Source: Vaccines (Basel). 2025 Jul 16;13(7):759. doi: 10.3390/vaccines13070759 (PMC12300279; doi:10.3390/vaccines13070759)
Supplement: Supplementary file 1 [file vaccines-13-00759-s001.zip › vaccines-3690447-supplementary.pdf]

vaccines

MDPI

Supplementary Materials

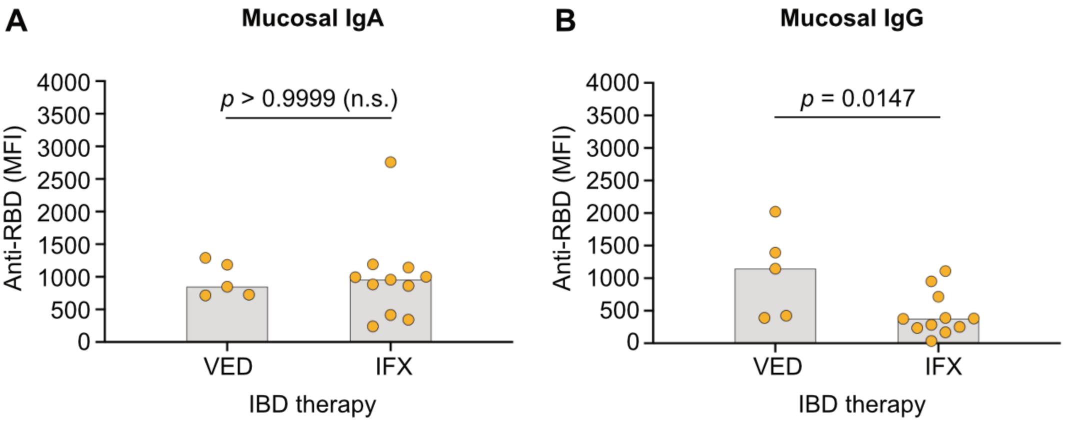

Supplementary figure S1. SARS-CoV-2 JN.1 variant-targeting mucosal immunity following vaccination with variant-adapted COVID-19 mRNA vaccines, stratified by IBD therapy (VED: vedolizumab; IFX: infliximab). Anti-receptor binding domain (RBD) IgA (A) and IgG (B) levels in saliva of patients with IBD presented as mean fluorescence intensity (MFI). Samples were collected two to four weeks after receiving a fourth vaccine dose with XBB.1.5 mRNA vaccines. Median values are indicated by bars. Statistical analyses are based on exact Mann-Whitney tests.

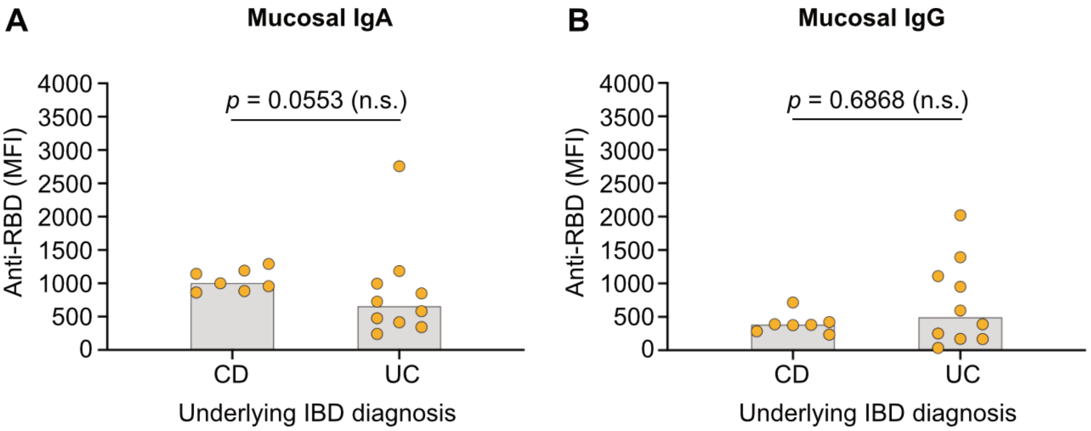

Supplementary figure S2. SARS-CoV-2 JN.1 variant-targeting mucosal immunity following vaccination with variant-adapted COVID-19 mRNA vaccines, stratified by IBD diagnosis (CD: Crohn's disease; UC: ulcerative colitis). Anti-receptor binding domain (RBD) IgA (A) and IgG (B) levels in saliva of patients with IBD presented as mean fluorescence intensity (MFI). Samples were collected two to four weeks after receiving a fourth vaccine dose with XBB.1.5 mRNA vaccines. Median values are indicated by bars. Statistical analyses are based on exact Mann-Whitney tests.

Table S1: STAR SIGN study investigators.

First name	Last name
Benjamin	Misselwitz[1,2]
Vasileios	Oikonomou[1]
Jacqueline	Wyss[1]
Niklas	Krupka[1]
Irina	Bergamin[3]
Reto	Bertolini[3]
Jan	Borovicka[3]
Remus	Frei[3]
Johannes	Haarer[3]
Rahel	Häuptle[3]
Pamela	Meyer-Herbon[3]
Mikael	Sawatzki[3]
Gian-Marco	Semadeni[3]
David	Semela[3]
Sarah	Zwyssig[3]

[1] Department of Visceral Surgery and Medicine, Inselspital Bern University Hospital, University of Bern, Bern, Switzerland

[2] Medical Clinic II, Ludwig Maximilian University of Munich, Munich, Germany

[3] Department of Gastroenterology and Hepatology, HOCH, Cantonal Hospital St. Gallen, 9007 St. Gallen, Switzerland
